# Can We Use Tree Rings of Black Alder to Reconstruct Lake Levels? A Case Study for the Mecklenburg Lake District, Northeastern Germany

**DOI:** 10.1371/journal.pone.0137054

**Published:** 2015-08-28

**Authors:** Ernst van der Maaten, Marieke van der Maaten-Theunissen, Allan Buras, Tobias Scharnweber, Sonia Simard, Knut Kaiser, Sebastian Lorenz, Martin Wilmking

**Affiliations:** 1 Institute of Botany and Landscape Ecology, University of Greifswald, Greifswald, Germany; 2 Climate Dynamics and Landscape Evolution, GFZ German Research Centre for Geosciences, Potsdam, Germany; 3 Staff Scientific Executive Board, GFZ German Research Centre for Geosciences, Potsdam, Germany; 4 Institute of Geography and Geology, University of Greifswald, Greifswald, Germany; Institute of Tibetan Plateau Research, CHINA

## Abstract

In this study, we explore the potential to reconstruct lake-level (and groundwater) fluctuations from tree-ring chronologies of black alder (*Alnus glutinosa* L.) for three study lakes in the Mecklenburg Lake District, northeastern Germany. As gauging records for lakes in this region are generally short, long-term reconstructions of lake-level fluctuations could provide valuable information on past hydrological conditions, which, in turn, are useful to assess dynamics of climate and landscape evolution. We selected black alder as our study species as alder typically thrives as riparian vegetation along lakeshores. For the study lakes, we tested whether a regional signal in lake-level fluctuations and in the growth of alder exists that could be used for long-term regional hydrological reconstructions, but found that local (i.e. site-specific) signals in lake level and tree-ring chronologies prevailed. Hence, we built lake/groundwater-level reconstruction models for the three study lakes individually. Two sets of models were considered based on (1) local tree-ring series of black alder, and (2) site-specific Standardized Precipitation Evapotranspiration Indices (SPEI). Although the SPEI-based models performed statistically well, we critically reflect on the reliability of these reconstructions, as SPEI cannot account for human influence. Tree-ring based reconstruction models, on the other hand, performed poor. Combined, our results suggest that, for our study area, long-term regional reconstructions of lake-level fluctuations that consider both recent and ancient (e.g., archaeological) wood of black alder seem extremely challenging, if not impossible.

## Introduction

Lake ecosystems appear as valuable sentinels of climate change, as they provide various direct and indirect indicators of change through the effects of climate [1]. Lake-level fluctuations of closed catchments, for example, reflect a dynamic water balance and provide detailed insight into past moisture variations. Data on lake-level fluctuations are generally obtained from instrumental gauging records (e.g., [2]) and reconstructions from sediments or landforms [3–5]. However, both these data sources have their limitations. Gauging records, for example, are often restricted to relatively short time periods. Geoscientific reconstructions, on the other hand, are highly demanding and require certain preconditions like the presence of dateable lacustrine sediments. Consequently, long-term information on lake-level fluctuations is lacking for many regions of the world. To extend lake-level series beyond the available instrumental records, tree rings of both recent and ancient (e.g., archaeological) wood may be considered an alternative natural proxy (e.g., [6–7]).

Tree-ring chronologies provide long-term, annually-resolved information on the growth performance of trees, which is known to vary with prevailing environmental conditions [8]. Hence, tree-ring chronologies have been used to reconstruct past environmental conditions including, for example, summer precipitation and temperature variability in Central Europe over the last 2500 years [9]. Also past hydrological fluctuations have been reconstructed using tree rings, for example for stream flow [10–11], basin water supply [12–13] or lake-level fluctuations [6, 14, 15]. Although mainly done for semi-arid regions with water availability as the main growth-limiting factor, dendrohydrological reconstructions were performed for other regions as well (cf. [16–17]).

In this study, we explore whether tree rings of black alder (*Alnus glutinosa* L.) can be used to reconstruct lake-level fluctuations for three lakes in the Mecklenburg Lake District, northeastern Germany. As gauging records for lakes in this area are typically available for time periods of 20 to 50 years only [18], long-term reconstructions could provide valuable information on past hydrological conditions. Such insights are crucial to increase the understanding on the dynamics of climate and landscape evolution in these northern Central European lowlands.

We selected black alder as our study species as this species is typically found as riparian vegetation along lakeshores (and rivers) due to a high tolerance against permanently waterlogged and temporally flooded conditions. As tree growth varies with environmental conditions, tree rings of lakeshore alder may capture information on past hydrological fluctuations, and may thus be used to reconstruct lake levels. This potential is also suggested by various dendroecological studies showing sensitivity of black alder growth to river flow (e.g., [19–20]) and groundwater fluctuations [21]. Although alder is a relatively short-lived tree species with a typical maximum age of about 120 years [22], the frequent use of its decay-resistant wood in foundations of historical buildings further offers the possibility of extending living tree chronologies back in time for several centuries [23].

**Fig 1:**
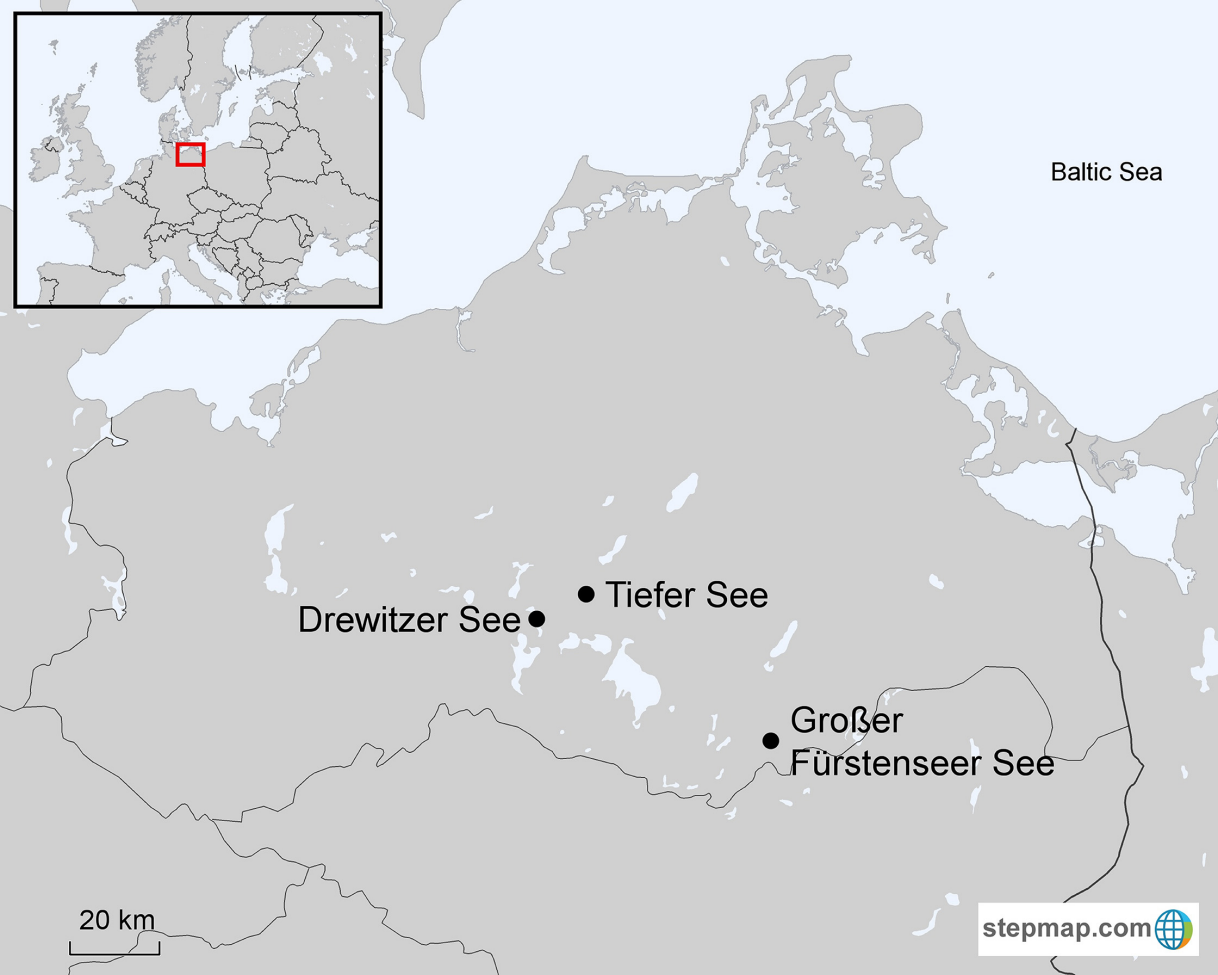
Location of the study lakes in northeastern Germany. An inset map shows the location of the study area within Europe.

Relationships between annual growth of black alder and lake-level fluctuations are studied for lakes Tiefer See, Drewitzer See and Großer Fürstenseer See (Fig 1). We hypothesized that (1) there is a common regional (i.e. climate-related) signal in (a) lake-level fluctuations and (b) tree growth of alder among the study lakes, and that (2) this regional signal can be used for lake-level reconstructions.

## Materials and Methods

### Study sites and tree-ring data

We sampled black alder stands surrounding the lakes Tiefer See, Drewitzer See and Großer Fürstenseer See, which are all located in the Mecklenburg Lake District in northeastern Germany (Fig 1). Research permissions were provided by the Nature Park Mecklenburgische Schweiz und Kummerower See (Basedow), Nature Park Nossentiner-Schwinzer Heide (Plau am See) and Müritz National Park (Hohenzieritz), respectively.

**Fig 2:**
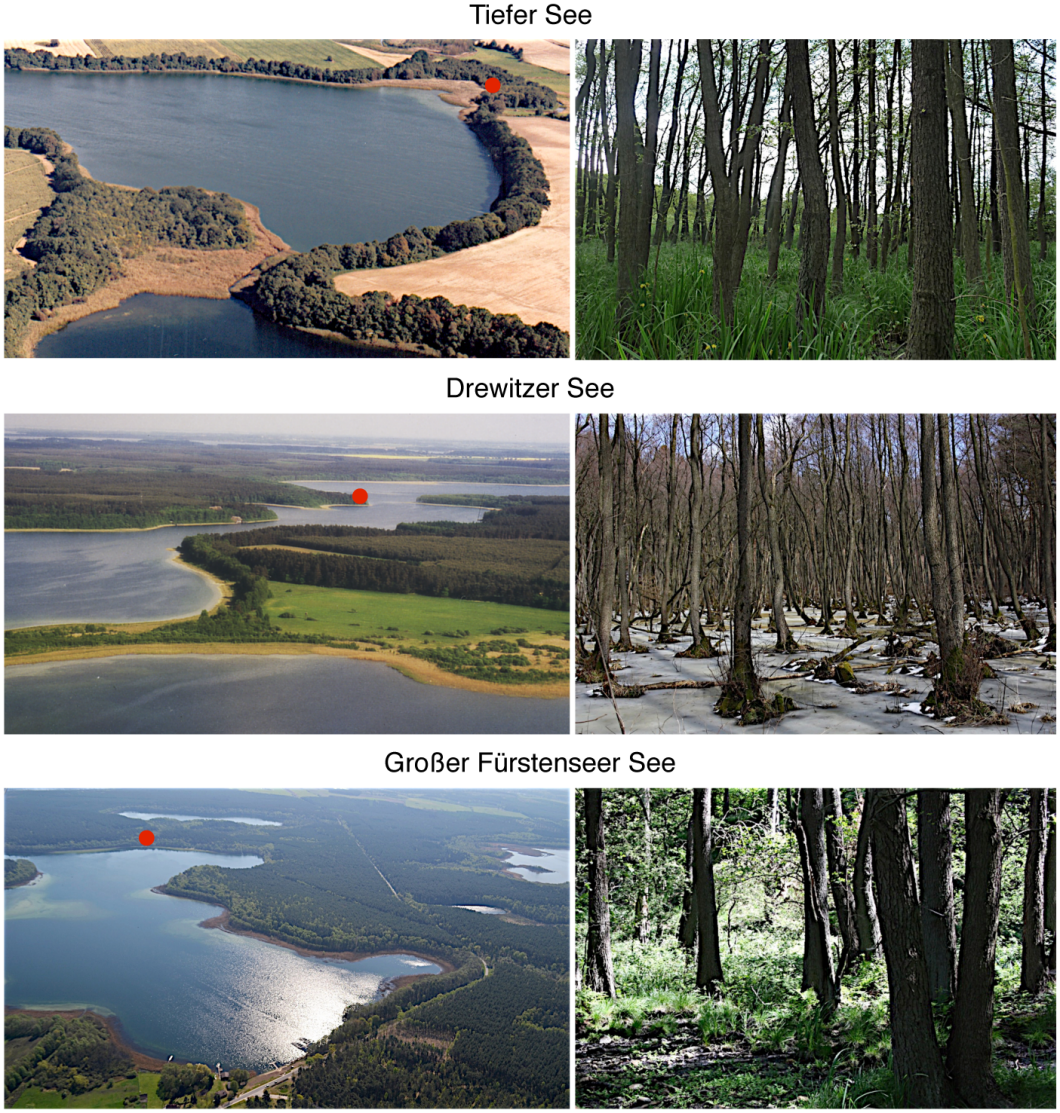
Photo compilation of the study lakes and lakeshore alder stands. On the left, oblique aerial views of the study lakes; red dots indicate approximate sampling locations. On the right, ground photos of the sampling sites. (Photos by Jörg Gast, Tobias Scharnweber and Peter Stüve).

Lake Tiefer See (lake area: 76 ha; catchment area: 550 ha; maximum depth: 62.5 m; location of sampling site: Lat 53.591, Long 12.536), which is steered by a weir, is part of the Klocksiner lake chain, and directly connected to lakes Hofsee and Flacher See. Lakes Drewitzer See (692 ha; 2,430 ha; 31.3 m; Lat 53.531, Long 12.369) and Großer Fürstenseer See (204 ha; 3,950 ha; 24.5 m; Lat 53.301, Long 13.173) have no or small, widely inactive surficial in- and outlets, respectively. The studied lakes all show a very narrow ratio between lake and catchment area (<1:10), and are therefore expected to show immediate and significant lake-level responses after precipitation [24]. Combined, these characteristics make that the studied lakes are particularly suitable for lake-level reconstructions. Black alder trees were sampled on homogeneous lake terraces within two meter above the current lake level on mineral (Drewitzer See) to organic soils (Tiefer See and Großer Fürstenseer See). As the alder stand at lake Tiefer See is located almost at the current lake level, this terrace was wettest. Photos from all study lakes and alder stands are presented in Fig 2; key parameters for the three lake ecosystems are summarized in S1 Table.

Per site, we selected ~20 dominant or co-dominant trees growing under similar microsite conditions (cf. sampling on homogenous lake terraces), and generally extracted two increment cores per tree at breast height (for an overview of the sample material see Table 1). Initially, cores were sanded with progressively finer grit sandpaper to highlight annual rings and to measure tree-ring widths (TRW) optically. However, as color variations in our alder samples complicated the optical measurement of TRW, we decided to change our procedure. Namely, we X-rayed the samples using an Itrax Multiscanner (Cox Analytical Systems, Sweden). Therefore, the increment cores were mounted on wooden holders and cut along their full length parallel to the transverse wood surface with a twin-blade saw (Walesch Electronic, Switzerland), producing 1.2 mm thick laths for the X-ray measurements. Ring boundaries were more distinct at the obtained gray-scale radiographic images (spatial resolution of 20 μm) compared to our optical scans (S1 Fig). Wood density measurements were not performed as several tests showed that no meaningful chronologies could be obtained (cf. Discussion). Measurements of TRW, as well as the consequent visual and statistical crossdating of tree-ring series were performed in CooRecorder v7.7 and CDendro (Cybis Elektronik and Data AB, Sweden).

**Table 1:** Characteristics of study trees and tree-ring chronologies.

Lake	# trees (cores)	Max. time span	Mean segment length (years)	Avg. ring width (mm)	RBAR	MS	AC	EPS
Tiefer See	20 (40)	1910–2011	68	1.77	0.45/0.48	0.35/0.35	0.21/0.58	0.94/0.95
Drewitzer See	19 (48)	1917–2012	85	1.37	0.41/0.28	0.29/0.29	0.42/0.66	0.93/0.88
Großer Fürstenseer See	23 (49)	1899–2013	93	1.47	0.53/0.47	0.32/0.32	0.36/0.69	0.96/0.95

Average ring width and chronology statistics for the detrended and standardized chronologies (detr/std) are presented for the common overlap period 1972–2011. *Max*. *time span* longest time period backboned by tree-ring data from at least one tree, *RBAR* mean inter-series correlation, *MS* mean sensitivity, *AC* first-order autocorrelation, *EPS* expressed population signal.

### Chronology development

We treated our tree-ring data in two different ways to allow for (1) analyses of high-frequency growth variations (i.e. climate sensitivity), and (2) reconstructions of lake-level fluctuations with chronologies that include low-frequency trends as well.

1. We detrended average ring-width series of individual trees by fitting a cubic smoothing spline with a 50% frequency cut-off at 30 years using MATLAB’s (V.7.9.0., R2009b) function *csaps* (V3.3.7) in combination with the spline smoothing parameter function *splinep* (presented courtesy of J.L. Dupouey). This detrending was done to remove long-term trends and to retain high-frequency variability [25]. Indices were then calculated by dividing the observed by the predicted values (S2 Fig).
2. We standardized individual tree-ring series by dividing TRW measurements by horizontal means to retain all low-frequency variability. Master chronologies for each site were then constructed by calculating a bi-weight robust mean of the standardized series (S3 Fig).

To assess the quality and character of the chronologies, we calculated the common chronology statistics mean inter-series correlation (RBAR), mean sensitivity (MS), autocorrelation (AC) and expressed population signal (EPS). RBAR is the average correlation coefficient between series, MS is a within-series statistic that measures the relative change in ring width from one year to the next, and AC is a measure of the previous year’s influence on current year’s growth [8]. EPS evaluates the confidence of the chronologies by indicating the degree to which the particular sample chronology portrays a hypothetically perfect chronology based on an infinite number of trees [26]. EPS values were calculated using the *wigley1* function (Tree-Ring MATLAB Toolbox, D. Meko).

### Environmental data

Monthly lake-level records of the three study lakes were available for the periods: 1985–2011—Tiefer See (readings of the directly connected lake Hofsee), 1983–2013—Drewitzer See, and 1974–2013—Großer Fürstenseer See (S4 Fig). In addition, a monthly groundwater record (1972–2013) was considered from an observation well at Klein Trebbow, close to lake Großer Fürstenseer See, that monitors the upper unconfined aquifer, hydraulically directly connected to the lake [2]. Lake-level and groundwater data were provided by the State Agency for Agriculture and Environment—Mecklenburg Lake District (StaLU-MS).

Site-specific climate data were obtained from a gridded climate surface of the German Weather Service (DWD), which is available from the web-based weather request and distribution system WebWerdis (http://www.dwd.de/webwerdis). This surface, based on the entire network of DWD-meteorological stations, has a high spatial resolution (1 x 1 km). Monthly air temperature records and precipitation sums were extracted for the period 1900–2013, and revealed, amongst others, that climate conditions are highly similar across the study lakes. The climate in the region can be characterized as temperate humid with a mean annual air temperature of 8.1°C and a mean annual precipitation sum of ~585 mm (as calculated over the Climate Normal Period 1961–1990; see for a climate diagram S5 Fig).

The temperature and precipitation data were used to calculate a Standardized Precipitation Evapotranspiration Index (SPEI), which is a drought indicator representing deviations from the average water balance (precipitation minus potential evapotranspiration; [27]). SPEIs were obtained with the SPEI package [28] in R [29] for time scales ranging from 6 to 72 months, representing the cumulative water balance over the previous *n* months [30]. Those different time domains are intended to represent drought conditions in different hydrological sub-systems, ranging from short-term fluctuations of soil water content to longer-term variations in groundwater storage [27].

### Statistical analyses

We performed a principal component analysis (PCA) on the detrended TRW series (cf. (1), section Chronology development) to explore high-frequency variations in the growth of individual trees. PCA is a data reduction method summarizing a data matrix (in this case: of individual tree-ring series) into a set of components, in such a way that the first component explains most of the variance in the dataset. In our analysis, the principal component *scores* represent common growth variation, whereas the component *loadings* indicate the association of individual tree-ring series with the component. The PC scores were correlated with monthly air temperature, precipitation and SPEI_6_ (SPEI integrated over 6 months, representing shorter-term fluctuations of soil water content) for May of the previous to September of the current year, to determine the climatic factors mainly responsible for high-frequency growth variations that were retained after detrending. Hence, this PCA allows for an assessment of possible differences in climate sensitivity of black alder from the different study lakes.

To reconstruct annual lake-level fluctuations for lakes Tiefer See and Drewitzer See, we built regression models with the master chronologies (cf. (2), section Chronology development). As the lake-level record of lake Großer Fürstenseer See showed various gaps, we reconstructed the strongly correlating parameter groundwater level (r = 0.84, p < 0.001) instead (cf. Fig 4 in [2]). In the reconstruction models, tree-ring width indices (RWI) for the years *t* and *t+1* were considered as predictors for water level in year *t* [14]. RWI in year *t+1* was included in the set of possible predictors to allow for lagged responses of tree growth to hydrological variations. For the study lakes, we tested all model variants, i.e., reconstruction models with RWI_t_, RWI_t+1_ and RWI_t_ + RWI_t+1_. Reconstructions are done for periods with at least 10 trees in the chronology, and a running EPS (30-year moving window) above the commonly used threshold of 0.85.

In addition to the tree-ring based reconstructions, we tested a second set of lake/groundwater level reconstruction models based on Standardized Precipitation Evaporation Indices. As SPEI represents a cumulative water balance (over a specified number of months; [30]), it likely captures information on variations in lake and groundwater level. We tested model variants with SPEI integrated over 1 to 6 years (12 to 72 months) to account for lagged responses of lake/groundwater level to weather conditions in preceding years. Reconstruction modeling was done in MATLAB.

In all reconstruction models, the variables lake level, groundwater level and SPEI were considered as averages over so-called *water years* from October of the previous year to September of the current year. A water year is hydrologically and ecologically more meaningful since part of the precipitation falling in late autumn and winter accumulates as snow and does not drain until snowmelt in spring.

## Results

**Fig 3:**
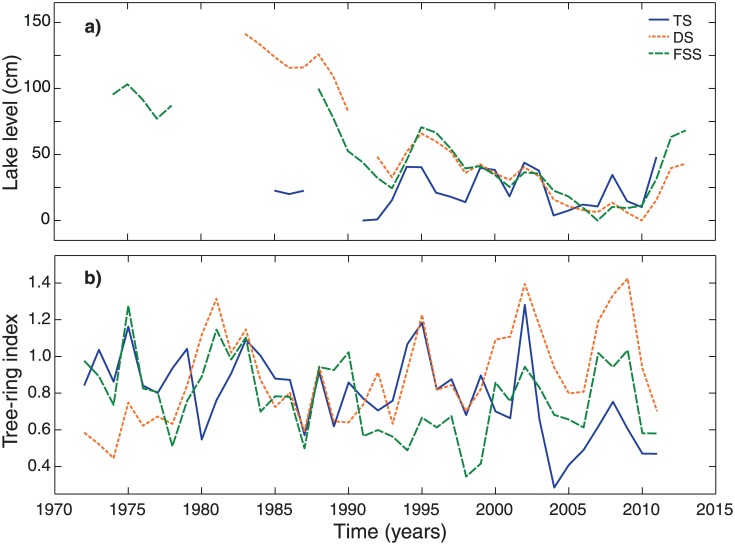
(a) Lake-level development and (b) tree-ring indices for the study lakes. For (a) years denote water years from October of the previous year to September of the current year. Standardized master chronologies are shown for the common overlap period of all trees (1972–2011). *TS* Tiefer See, *DS* Drewitzer See and *FSS* Großer Fürstenseer See.

A comparison of lake-level records reveals differences between the study lakes. At low frequency, for example, a negative lake-level trend is observed at lakes Drewitzer See and Großer Fürstenseer See from approximately 1988 to 2010. This negative trend is most pronounced for lake Drewitzer See, and absent at lake Tiefer See (Fig 3a). At high frequency, on the other hand, lake Tiefer See is characterized by larger yearly amplitudes of lake-level fluctuations (S4 Fig). These findings suggest that local conditions (e.g., characteristics of the catchment area) more strongly affect lake levels of the study lakes than regional conditions (e.g., regional climate) do.

The importance of local conditions is also reflected in the master chronologies of lakeshore alder, as indicated by distinct growth patterns (Fig 3b). Cross-correlations are low and only significant between chronologies from DS and FSS (r_TS-DS_ = 0.03, r_TS-FSS_ = 0.25, r_DS-FSS_ = 0.37; calculated over the period 1972–2011).

Chronology statistics for the detrended and standardized tree-ring series, as calculated over the common overlap period of all trees (1972–2011), are provided in Table 1. Relatively high RBAR-values indicate that trees from the same site show similar growth patterns. MS-values range between 0.29 and 0.35, pointing to high sensitivity of alder growth to environmental conditions. High EPS values suggest strong common signals in the obtained chronologies.

**Fig 4:**
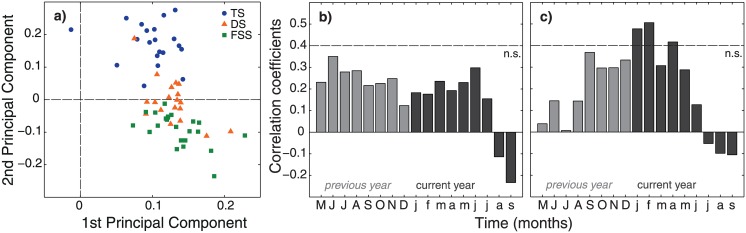
(a) PCA biplot and Pearson correlation coefficients of (b) PC1 and (c) PC2 scores with monthly SPEI_6_. The biplot shows the loadings of 62 detrended tree-ring series with the first and second principal component. Symbols represent tree-ring series for the different study lakes. Pearson correlations are shown for May of the previous to September of the current year. The significance level of the correlations (p < 0.05) is indicated by dotted lines. The PCA was performed over the common overlap period 1972–2011. SPEI_6_ data for lake Tiefer See were used.

The first and second principal component (PC) of the principal component analysis explained 46.6% of the total variance in the dataset (33.6 and 13.0%, respectively). Associations of the individual tree-ring series with the PC scores show that all trees (except for one) have a positive loading on PC1, whereas PC2 roughly separates the trees from lake Tiefer See from those of lake Großer Fürstenseer See (see biplot Fig 4a). Correlations of the PC1 and PC2 scores with monthly air temperature and precipitation data were largely non-significant (S6 Fig). For the integrative climate parameter SPEI_6_, correlations with PC1 scores were all non-significant (Fig 4b), whereas correlations with the less explaining PC2 scores were only significant for January, February and April of the current year (Fig 4c).

Combined, the results on lake-level fluctuations (Fig 3a and S4 Fig) and growth of alder (Figs 3b and 4) stress that local signals prevail over a regional signal. Hence, we had to reject our first hypothesis and rephrase our second one into: local signals in tree growth can be used for lake-level reconstructions of individual lakes.

**Fig 5:**
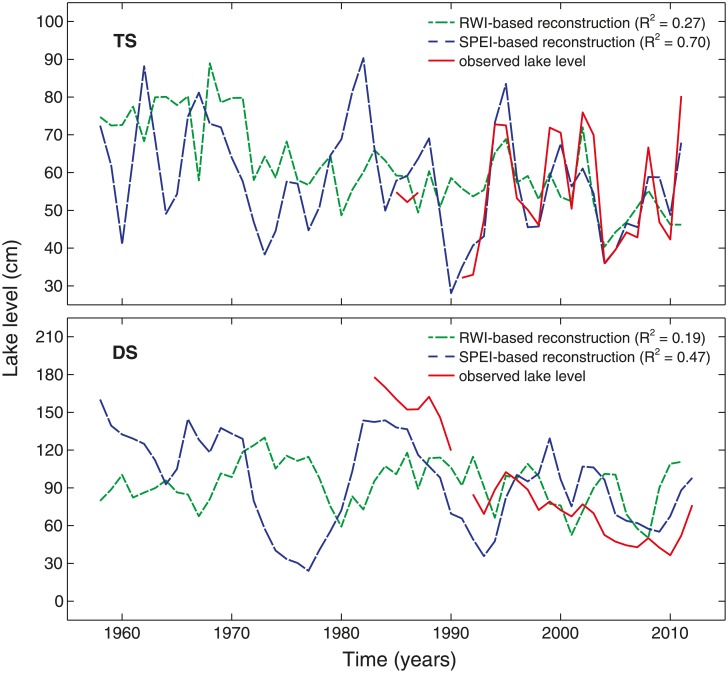
Reconstructed lake-level fluctuations for lakes Tiefer See and Drewitzer See. Reconstructions are based on RWI and SPEI parameters and compared with the observed lake level. They are shown for the period 1959–2011/2012 (period with ≥ 10 trees in the chronology; running EPS (30-year moving window) above the commonly used threshold of 0.85). Years denote water years from October of the previous year to September of the current year. Model equations are provided in Table 2.

**Fig 6:**
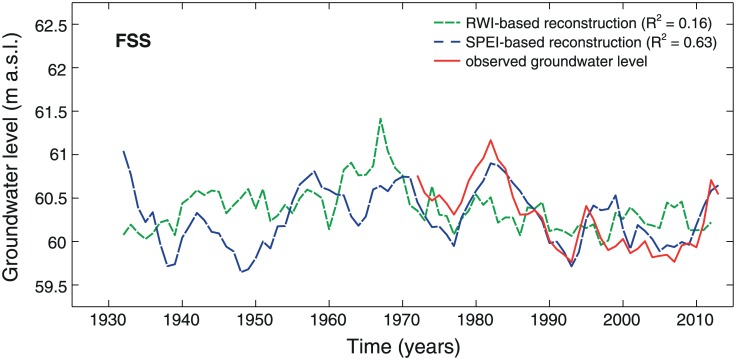
Reconstructed groundwater-level fluctuations for lake Großer Fürstenseer See. Reconstruction period 1932–2013. Further details in legend of Fig 5.

We built reconstruction models for lake-level and groundwater fluctuations using a predictor pool of either tree-growth or SPEI parameters (see section Statistical analyses). For all lakes, SPEI-based reconstruction models had a relatively high explained variance (R^2^-values ranging between 0.47 and 0.70; Table 2) and were able to reflect the magnitude of the observed fluctuations (Figs 5 and 6). The best performing models included the parameters SPEI_24_ for lake Tiefer See and SPEI_72_ for lakes Drewitzer See and Großer Fürstenseer See. Tree-ring based models, on the other hand, displayed low R^2^-values ranging between 0.16 and 0.27. The explanatory variables differed between the study lakes (TS: RWI_t_, DS and FSS: RWI_t+1_).

**Table 2:** RWI- and SPEI-based reconstruction models of lake (LL) and groundwater level (GW) for the study lakes.

Lake	Model equation	R^2^
Tiefer See	LL = 31.7834 x RWI_t_ + 31.3010	0.27**
	LL = 14.4390 x SPEI_24_ + 60.0453	0.70***
Drewitzer See	LL = -81.3328 x RWI_t+1_ + 166.1264	0.19*
	LL = 36.8243 x SPEI_72_ + 100.8282	0.47***
Großer Fürstenseer See	GW = 0.7238 x RWI_t+1_ + 59.7108	0.16*
	GW = 0.3564 x SPEI_72_ + 60.3187	0.63***

Asterisks denote significance levels of the regression models:

* p < 0.05

** p < 0.01

*** p < 0.001.

## Discussion and Conclusion

Lake-level fluctuations of the study lakes showed distinct courses with differences both in longer-term trends (Fig 3a) as well as in the magnitude of yearly lake-level amplitudes (S4 Fig). The most pronounced was a negative lake-level trend observed for the distantly located lakes Drewitzer and Großer Fürstenseer See (Fig 1). This negative trend, also reported for other lakes in the Mecklenburg Lake District, is suggested to be caused by decreasing groundwater recharge, resulting from specific weather conditions in the last decades and high transpiration rates of dominating Scots pine plantations in the lake catchments [2, 31, 32]. Specific hydrological and catchment characteristics of lake Tiefer See, i.e. the lake level is steered by a weir and is mainly surrounded by agricultural land, may cause that no such trend is observed here, and that yearly lake-level amplitudes are larger (due to relatively high surface runoff). A shorter response time of lake Tiefer See is also reflected in lower AC-values of black alder tree-ring series (Table 1), and the inclusion of SPEI_24_ in the SPEI-based reconstruction model (rather than SPEI_72_ for the other lakes; Table 2).

Next to the lake-level fluctuations, also growth of black alder showed site-specific signals (Figs 3b and 4a), which could-at high-frequency- not be related to a specific climatic factor (Fig 4b and S6 Fig). Hence, lake-level reconstruction models were built for the study lakes individually (Figs 5 and 6). The SPEI-based models performed statistically well (Table 2; Figs 5 and 6). However, the reliability of the SPEI-based reconstructions might be questioned for the period that is not backboned by instrumental gauging records. Namely, the Standardized Precipitation Evapotranspiration Index is based on climatic input variables only (i.e. air temperature and precipitation), and can therefore not account for any human influence on hydrological conditions. Although this may not be an issue for natural lake ecosystems, severe problems may arise when catchment areas are affected by man. The latter applies to the lakes considered in this study, as they were and are exposed to human impacts on groundwater recharge through land use (e.g., extensive planting of Scots pine) and to surficial drainage by small artificial streams [2, 33, 34].

The tree-ring based models, on the other hand, showed a poor performance as indicated by R^2^-values ranging between 16 and 27% for the individual lakes (Table 2; Figs 5 and 6). Although other studies, considering tree-ring networks of (multiple) tree species from various locations within catchment areas, could report higher R^2^-values (36–50% variance explained; [6, 14, 15, 17]), even these reconstructions suggest that lake-level fluctuations and tree growth are only loosely coupled [6]. One possible explanation could be the complexity of lake ecosystems. Namely, multiple internal feedbacks and characteristics of the catchment, i.e., geographic location, regional climate, groundwater level, vegetation and land use [1, 35], affect the behavior of lakes. Hence, response times and magnitudes of lake-level fluctuations may strongly differ, resulting in complex interactions with the growth of riparian vegetation. Precipitation anomalies, for example, may have direct effects upon the growth of black alder, but also indirect through lagged effects upon lake level. Further, lake levels may be affected by directional shifts in precipitation over many years [36]. And although the aforementioned precipitation anomalies and directional shifts may result in comparably high lake levels, growth responses of alder may be highly distinct, varying between enforced growth responses to sudden lake-level extremes and adaptive responses towards slowly changing environmental conditions.

The hypothesis on sudden versus adaptive growth responses questions the applicability of a linear modeling approach to display relationships between lake-level fluctuations and tree growth. The assumption of linearity is furthermore impeded by the possibility of observing comparable growth levels with contrasting lake levels. In dry years, for example, alder may build narrow rings since its roots are known to be vulnerable to drought [37]. On the other hand, high water levels (or floods) may subject alder to stresses that lead to narrow rings as well [19–21], e.g., by inducing hypoxic conditions that constrain tree growth [38]. In addition, differences in substrate (organic vs. mineral) may influence alder growth at the study lakes.

Overall, our results suggest that long-term regional reconstructions of lake-level fluctuations in the Mecklenburg Lake District using tree rings of black alder seem extremely challenging, if not impossible, as a major common signal in both lake-level and tree-growth variations was lacking. Further, tree-ring based reconstructions for individual study lakes performed poorly, and, together with the near impractical task of extending lake-specific living tree chronologies back in time using archaeological wood from that specific lake (i.e. if not preserved as stems in the same lake, exact origins are usually unknown), this hampers long-term lake-level reconstructions as well. In future studies, reconstructions for individual lakes might be improved by including other tree-ring proxies like wood anatomical characteristics (e.g., vessel size) or stable isotopes [39–40]. Alternatively, it might be advantageous to consider a network containing ring-width chronologies of multiple tree species from throughout the catchment area (e.g., [6, 13, 14]) rather than lakeshore alder only. Statistically, filtering or smoothing, e.g., of reconstructed time series, might increase the correlation of observed and reconstructed lake-level data [14]. However, such a procedure does not account for any non-linear growth responses of alder to hydrological changes either. Non-linear models, although not suitable for reconstruction purposes, may nevertheless help to elucidate growth determinism for the period for which instrumental data is available.

The ecological understanding on growth determinism of alder may further be improved by studying TRW in conjunction with other tree-ring parameters, whereby not only wood anatomical parameters, but also the analysis of tree-ring stable isotopes seems promising. Namely, the analysis of stable isotopes gives insight in tree physiological responses, with a dual analysis of carbon and oxygen isotopes allowing the distinction between stomatal and photosynthetic responses [41]. Furthermore, wood density profiles may be investigated, as observed intra-annual density fluctuations in black alder [42] might reflect changes in hydrologically extreme years [8]. On the contrary, establishing inter-annual wood density chronologies, for example of maximum latewood density (MXD), seems less promising for alder. In some preliminary analyses, we namely observed that such chronologies contain only weak common signals, likely due to macroscopic characteristics of black alder wood. Its very thin latewood (cf. S1 Fig), for example, hampers the development of meaningful MXD chronologies. Another possibility to increase the understanding on hydrological forcing of black alder growth is by studying lakeshore alder at lakes with longer instrumental lake-level records available. Longer records likely better reflect the full range of possible lake levels, and may thus be used to explore the relationship between lake level and tree growth in more detail, to detect possible threshold behavior and to assess interactions with temperature and precipitation in particular periods, i.e. in dry or wet episodes [43].

## Supporting Information

S1 Fig Example of an optical scan (*top*) and an X-ray scan (*bottom*) of black alder. Scans display the outer tree rings (9 full rings) for two samples of a single tree at lake Tiefer See.
(EPS)

S2 Fig Detrended tree-ring series for the study lakes. Gray lines represent index values for individual trees, black lines average series. *TS* Tiefer See, *DS* Drewitzer See and *FSS* Großer Fürstenseer See.
(EPS)

S3 Fig Standardized tree-ring series for the study lakes. Gray lines represent index values for individual trees, black lines master chronologies. *TS* Tiefer See, *DS* Drewitzer See and *FSS* Großer Fürstenseer See.
(EPS)

S4 Fig Lake-level development for the study lakes. Monthly lake-level records for lakes Tiefer See (TS), Drewitzer See (DS) and Großer Fürstenseer See (FSS).
(EPS)

S5 Fig Climate diagram representative for the study region. Mean courses of monthly air temperature (in °C; black line) and monthly precipitation (in mm; gray bars) are shown for the climate normal period 1961–1990 (based on data for lake Tiefer See).
(EPS)

S6 Fig Pearson correlation coefficients of PC1 and PC2 scores with monthly temperature (*top*) and precipitation data (*bottom*). Pearson correlations are shown for May of the previous to September of the current year. The significance level of the correlations (p < 0.05) is indicated by dotted lines.
(EPS)

S1 Table Key parameters of lake ecosystems. *MAT* mean annual air temperature, and *MAP* mean annual precipitation as calculated from the gridded DWD data for the climate normal period 1961–1990; *AP range* annual precipitation range over the climate normal period.
(DOCX)
