# Supplementary material for: Can We Use Tree Rings of Black Alder to Reconstruct Lake Levels? A Case Study for the Mecklenburg Lake District, Northeastern Germany
Source: PLoS One. 2015 Aug 28;10(8):e0137054. doi: 10.1371/journal.pone.0137054 (PMC4552791; doi:10.1371/journal.pone.0137054)
Supplement: S1 Table — MAT mean annual air temperature, and MAP mean annual precipitation as calculated from the gridded DWD data for the climate normal period 1961–1990; AP range annual precipitation range over the climate normal period. (DOCX) [file pone.0137054.s007.docx]

| **Parameters** | **Studied lake ecosystems** | | |
| --- | --- | --- | --- |
|  | Tiefer See | Drewitzer See | Großer Fürstenseer See |
| Lake ID | TS | DS | FSS |
| Latitude (°) | 53.591 | 53.531 | 53.301 |
| Longitude (°) | 12.536 | 12.369 | 13.173 |
| Altitude (m a.s.l.) | 62.7 | 61 | 63.7 |
| In-/outflow | permanent | no | periodic |
| Lake area (ha) | 75.7 | 691.8 | 204 |
| Max. lake depth (m) | 62.5 | 31.3 | 24.5 |
| Lake-level gauging since (year) | 1984 | 1982 | 1973 |
| Max. lake-level amplitude (cm) | 111 | 147 | 134 |
| Trophic state | mesotrophic | eutrophic | mesotrophic |
| Catchment area (ha) | 550 | 2430 | 3950 |
| Catchment geology (prevailing) | ice marginal zone | outwash plain | outwash plain |
| Catchment land-cover (prevailing) | agricultural land | coniferous forest | coniferous forest |
| Reference | Kienel et al. 2013; J Paleolimn 50:535-544 | [34] | [2] |
| MAT (°C) | 8.0 | 8.1 | 8.1 |
| MAP (mm) | 584 | 582 | 587 |
| AP range (mm) | 422-795 | 392-799 | 426-749 |
